# Association of SSRI and SNRI use with incidence of cardiovascular events in veterans with giant cell arteritis and polymyalgia rheumatica

**DOI:** 10.3389/fimmu.2025.1509941

**Published:** 2025-04-24

**Authors:** Tianyu Zhang, Chris A. Gentry, Nicole M. Kuderer, Gary H. Lyman, Bernard Ng, Despina Michailidou

**Affiliations:** ^1^ Department of Statistics and Data Science, Carnegie Mellon University, Pittsburgh, PA, United States; ^2^ Pharmacy Service, Oklahoma City VA Health Care System, Oklahoma City, OK, United States; ^3^ Advanced Cancer Research Group, Kirkland, WA, United States; ^4^ Department of Medicine, Duke University School of Medicine, Durham, NC, United States; ^5^ Public Health Sciences and Clinical Research Divisions, Fred Hutchinson Cancer Center, Seattle, WA, United States; ^6^ VA National Rheumatology Program, Lexington, KY, United States; ^7^ Division of Rheumatology, University of Oklahoma Health Sciences Center, Oklahoma City, OK, United States; ^8^ Division of Rheumatology, Oklahoma City VA Health Care System, Oklahoma City, OK, United States

**Keywords:** giant cell arteritis, polymyalgia rheumatica, SSRI, SNRI, ischemic stroke, TIA, myocardial infarction, angina

## Abstract

The leading cause of death in patients with giant cell arteritis (GCA) and polymyalgia rheumatica (PMR) is cardiovascular disease. The objective of this study was to determine whether the use of selective serotonin reuptake inhibitors (SSRI) and serotonin norepinephrine reuptake inhibitors (SNRI) in veterans with GCA and PMR could have a cardio-modulatory effect as compared to nonuse. Patients with GCA and PMR were identified through the Veterans Affairs Informatics and Computing Infrastructure. After a 2:1 propensity score matching for SSRI or SNRI users, we identified nonusers with similar covariates. We then applied a multivariate logistic regression (MLR), to calculate the odds ratio (OR) for cardiovascular event (CVE) outcomes within 5 years after the index date. Related hazard ratios (HR) were also calculated to validate the discovery of our findings. We identified 2249 patients with GCA and 3906 patients with PMR. Among patients with GCA, 174 (27%) SSRI users had incident cardiovascular disease as compared to 47 (28%) SNRI users and 277 (19%) nonusers; in the PMR cohort, 108 (13%) were SSRI users compared to 71 (15%) SNRI users and 255 (11%) nonusers. The adjusted ORs of the CVE outcome associated with venlafaxine (2.44, p=0.01) and sertraline (1.45, p=0.04) were significantly greater than 1 in GCA, with similar results observed in the PMR cohort (2.01, p=0.02, and 1.45, p=0.04, respectively). Cox-regression analysis was also conducted, and the hazard ratios were qualitatively consistent with the MLR analysis. In conclusion, the adjusted risk of CVE in patients with GCA or PMR using either venlafaxine or sertraline was higher than that in the non-exposed groups.

## Introduction

Giant cell arteritis (GCA) and polymyalgia rheumatica (PMR) are chronic idiopathic inflammatory conditions of unclear etiology (1) with significant implications for cardiovascular morbidity and/or mortality. Both conditions are characterized by increased activated platelet activation, with circulating levels of the platelet marker thrombospondin-1 (TSP-1) recently demonstrated to be significantly higher in patients with GCA and PMR as compared to healthy controls (2–4). Activated platelets undergo degranulation resulting in the release of dense and alpha granules that contain serotonin and other platelet activating factors (5). Upon interaction with other platelets, they contribute to coagulation promoting atherosclerosis that is complicated by arterial thrombosis, myocardial infarction, and ischemic stroke (6). Patients with GCA are at higher risk of developing cardiovascular events (CVE) compared to non-GCA patients, with the highest risk of these events occurring within the first year of GCA diagnosis (7). A recent study demonstrated that patients with GCA experienced myocardial infarct (MI) that was mainly type 2 due to systemic inflammation (8). However, in other studies, there was no increased risk of acute coronary syndrome in GCA (9). Patients with PMR also have a higher risk of cardiovascular events, with that risk being greatest in patients who are younger than 60 years at the time of diagnosis (10).

Selective serotonin reuptake inhibitors (SSRI) and serotonin norepinephrine reuptake inhibitors (SNRI) could have a beneficial effect on the cardiovascular system by inhibiting platelet aggregation and therefore reducing cardiovascular mortality and morbidity (11). Some of the newest SSRI such as sertraline, can improve insulin resistance, dyslipidemia, and have anti-inflammatory action reducing C-reactive protein and interleukin-6 (12). Duloxetine, an SNRI was found to have antiplatelet and thrombo-protective properties (13). Studies investigating the risk of primary ischemic events among SSRI users have been inconclusive. Because SSRI inhibit serotonin uptake into platelets, there could be a reduction in the risk of ischemic heart events (14–16). In a randomized, double-blind placebo-controlled trial that recruited patients with depression within 30 days of hospitalization for acute MI or unstable angina, patients were randomly assigned to receive either sertraline or a placebo for 24 weeks. Overall, severe CVE was less with sertraline compared to placebo (14.5% vs 22.4%) though not statistically significant (17). Other studies have shown no difference in the cardiovascular risk in depressed patients treated with SSRI compared to non-depressed patients who did not use any anti-depressants (18).

The incidence and risk of cardiovascular events in patients with GCA and PMR who use SSRI and SNRI have not been carefully investigated. The objective of our retrospective study was to evaluate the association of SSRI or SNRI users with cardiovascular outcomes defined to include ischemic stroke, transient ischemic attack (TIA), myocardial infarction, and angina in patients with GCA and PMR within a veteran-based population.

## Methods

### Data sources and study population

In this retrospective study approved by our institutional review board, patients with GCA and PMR, were identified between January 1999 and September 2023, and extracted through the Veterans Affairs Informatics and Computing Infrastructure (VINCI). Cardiovascular events were identified using diagnostic codes to search hospital admissions and outpatient visits within the Veterans Health Administration system, and linkage with the Centers for Medicare and Medicaid administrative data, as previously performed by our group (19, 20). VINCI also provides information about outpatient pharmacy dispensing that allowed us to identify SSRI (citalopram, escitalopram, fluoxetine, paroxetine, sertraline, vortioxetine, and fluvoxamine) and SNRI (duloxetine, venlafaxine, desvenlafaxine, milnacipran) users among GCA and PMR patients.

### Study design and data collection

Matched cohort analyses were conducted for incident CVE among patients with GCA or PMR who used SSRI or SNRI, and findings were compared to patients with GCA or PMR who did not use any anti-depressants. The comparison cohort was created by matching two individuals with GCA or PMR who did not use SSRI or SNRI to each GCA or PMR patient who used SSRI or SNRI within 6 weeks before diagnosis of GCA or PMR with total use of at least 365 days. Initiation of SSRI or SNRI was confirmed by more than one filled prescription of either brand or generic drug names. We included in our database patients who had been on other antidepressants such as clomipramine, amoxapine, desipramine, trazodone, doxepin, imipramine, phenelzine, mirtazapine, bupropion, bupropion/naltrexone, or clomiphene prior to the index date (date of GCA or PMR diagnosis). The matching was performed based on gender, age at the time of disease diagnosis, race, glucocorticoid use for at least 150 days, hypertension (HTN), smoking, diagnosis of vasculitis or PMR in the outpatient or inpatient setting, and average Charlson’s comorbidity score 5 years prior to the index date.

The GCA cohort included patients: (a) aged ≥50 years; (b) classified with at least one GCA clinical modification code from the International Classification of Diseases Ninth or Tenth Revision (ICD-9-CM or ICD-10-CM) (**Supplementary Table 1**), and (c) on glucocorticoid therapy and/or other immunosuppressive therapy for at least 150 days, starting within 30 days before diagnosis or within 365 days after diagnosis. Similarly, the PMR cohort included patients: a) aged ≥ 50 years; b) classified with the ICD-9-CM or ICD-10-CM codes for PMR (**Supplementary Table 1**), and (c) on glucocorticoid therapy and/or other immunosuppressive therapy for least 150 days, starting within 1 month prior to diagnosis or within the first year of diagnosis. Identification of patients with GCA and PMR by using ICD-CM codes and administrative databases has similarly been applied in the literature before (19–22). Our study participants were followed up until the date of a cardiovascular outcome (ischemic stroke, TIA, myocardial infarction, angina), the end of the 5-year observation period, death, or the end of our study (30 September 2023), whichever happened first.

### Study outcome of interest

The outcome of interest was the first incidence of cardiovascular events (ischemic stroke, TIA, myocardial infarction, angina) after the index date among the study cohorts. Cardiovascular events were defined by using ICD-9 and ICD-10 procedural codes (**Supplementary Table 1**). We included patients who had a prior history of cardiovascular events for at least 12 months prior to the index date. However, we excluded patients who had a prior history of cardiovascular events and had been on dual anti-platelet therapy (aspirin plus clopidogrel, aspirin plus dipyridamole, aspirin plus prasugrel, or aspirin plus ticagrelor) for at least 6 months prior to the index date. We also excluded patients who had deep venous thrombosis and/or pulmonary embolism and had been on heparin, warfarin, apixaban, dabigatran, edoxaban, or rivaroxaban within 6 months prior to the index date.

### Use of covariates

Covariates were considered potential confounders that are recognized as independent risk factors for cardiovascular events, including, HTN, body mass index (BMI), and smoking. HTN was identified using the ICD-9 and ICD-10 codes (**Supplementary Table 1**) and assessed at baseline. Baseline BMI and smoking status prior to the start of the disease cohort follow-up were also included as covariates. BMI is known to be a confounder for cardiovascular events (23), whereas smoking is a well-characterized risk factor for cardiovascular disease (24). Basic demographic information such as age, gender, race/ethnicity, and information about the initial diagnosis of GCA in the outpatient or inpatient setting were also included as potential confounders. Patients with malignancy were excluded from this study after identifying at least two encounters for ICD-9 or ICD-10 codes for malignancy within 6 months prior to the index date, because of the association between heart disease and malignancy (25). As a comorbidity index score, we used the Charlson comorbidity score that was calculated based on 19 medical conditions including diabetes, heart disease, and cancer using data up to 5 years prior to the index date (26).

### Statistical analysis

Baseline demographic characteristics were detailed based on the stratification with or without SSRI or SNRI use for the GCA and PMR cohorts. Mean ± standard deviation (SD) for quantitative variables is reported, whereas for categorical variables, proportions expressed as a percentage (based on non-missing values) are presented. Descriptive statistics for the frequency of CVE were also calculated after matching based on the same stratification among the two cohorts. After performing a 2:1 propensity score matching for confounding control, we identified pairs of SSRI and/or SNRI users as well as nonusers among the two patient cohorts and applied a multivariate logistic regression (MLR) over the matched data, to calculate the odds ratio (OR) for any CVE outcome within 5 years after the date of cohort entry (index date) in each SSRI or SNRI medication.

Multivariable Cox hazard regression models were also implemented to assess hazard ratios (HR) and the estimates are reported with 95% confidence intervals (CIs). The censoring events in the time-to-event analysis were death, end of the study (30 September 2023), and malignancy that some of the patients might have developed during their disease. Multivariable Cox regression analysis was adjusted for BMI, gender, age at time of disease diagnosis, race, smoking, HTN, disease diagnosis in the outpatient or inpatient setting, and 5-year average Charlson’s score.

For all analyses, *p*-values < 0.05, were considered statistically significant. All statistical analyses were performed using the R software (version 3.6.3, http://www.r-project.org/).

## Results

### Baseline demographic characteristics of the study populations

After matching, a total of 2249 patients with GCA and 3906 patients with PMR were included in our study. Within the GCA cohort, 653 were SSRI users, 168 were SNRI users, and 1428 were nonusers. Among the PMR cohort, 815 and 487 were SSRI and SNRI users respectively, whereas 2604 were nonusers. Most of the patients were male and white in both cohorts. The mean age of patients with GCA at the time of diagnosis ranged between 68 to 74 years, whereas in the PMR cohort was above 70 years (**Table 1**). Less than one-fourth of the patients were smokers both in the GCA and PMR cohorts. The mean BMI ranged between 28 to 30 in the GCA and PMR cohorts. The frequency of HTN, which is a known risk factor for cardiovascular events (27) was higher in patients with GCA and was observed in more than half of the patients. A possible explanation for this observation is that patients with GCA are usually treated with higher doses of glucocorticoids as compared to patients with PMR.

**Table 1:** Baseline demographic characteristics of SSRI, SNRI and nonusers in the GCA and PMR cohorts. .

GCA, N= 2249 PMR, N=3906						
	Nonusers N=1428	SSRI users N=653	SNRI users N=168	Nonusers N=2604	SSRI users N=815	SNRI users N=487
Age, years (mean, IQR)	74 (50-97)	71 (50-97)	68 (51-96)	72 (50-97)	72 (50-97)	71 (50-97)
Sex (male), N (%)	1339 (94%)	603 (92%)	149 (89%)	2506 (96%)	764 (94%)	444 (91%)
Race/Ethnicity, N (%)						
White	1017 (71%)	471 (72%)	125 (74%)	2184 (84%)	669 (82.08%)	398 (82%)
African American	142 (10%)	74 (11%)	19 (11%)	144 (5.5%)	46 (5.64%)	41 (8%)
Native American	12 (0.9%)	4 (0.5%)	1 (0.6%)	21 (0.5%)	8 (0.98%)	2 (0.4%)
Hispanic or Latino	61 (4%)	23 (3.5%)	7 (4%)	76 (3%)	27 (3.31%)	17 (3.5%)
Asian	2 (0.1%)	0 (0%)	1 (0.6%)	18 (0.5%)	3 (0.37%)	1 (0.2%)
Unknown	178 (13%)	75 (12%)	15 (9%)	144 (5.5%)	52 (6.38%)	22 (4.5%)
Native Hawaiian	16 (1%)	6 (1%))	0 (0%)	27 (1%)	10 (1.22)	6 (1.2%)
Smoking, N (%)	262 (18%)	131 (20%)	41 (24%)	430 (17%)	146 (18%)	78 (16%)
BMI, (mean, IQR)	27.93 (14.26-58.71)	28.93 (15.37-62.10)	30.34 (16.23-51.50)	29.05 (10.04-61.76)	29.69 (17.60-60.71)	30.47 (17.07-53.08)
Charlson (mean, IQR)	4.68 (0.4-14.2)	4.75 (0.2-16.2)	4.9 (0.4-13.6)	4.64(0.2-15.4)	4.84 (0.2-13.4)	4.75 (0.2-13)
HTN, N (%)	846 (59%)	430 (66%)	77 (46%)	1141(44%)	396 (48%)	198 (41%)
Steroid use, N (%)	1374 (96%)	636 (97%)	157 (93%)	2603 (99.9%)	815 (100%)	487 (100%)
Other IMT, N (%)	8 (0.6%)	23 (3%)	14 (8%)	1 (0.1%)	0 (0%)	0 (0%)
Dx as outpatient, N (%)	1260 (88%)	557 (85%)	142 (85%)	2504 (96%)	778 (95%)	459 (94%)
Ischemic stroke, N (%)	43 (3%)	38 (6%)	6 (4%)	8 (0.3%)	5 (1%)	6 (1%)
TIA, N (%)	104 (7%)	59 (9%)	13 (8%)	76 (3%)	27 (3%)	13 (3%)
MI, N (%)	88 (6%)	39 (6%)	15 (9%)	87 (3%)	45 (5%)	26 (5%)
Angina, N (%)	101 (7%)	80 (12%)	23 (14%)	127 (5%)	51 (6%)	38 (8%)
Prior CV event, N (%)	63 (4%)	40 (6%)	10 (6%)	53 (2%)	12 (2%)	11 (2%)
Anti-coag w6m, N (%)	144 (10%)	57 (8%)	17 (10%)	232 (9%)	88 (11%)	65 (13%)
Dual aPLT w6m, N (%)	105 (7%)	68 (10%)	17 (10%)	121(5%)	56 (7%)	19 (4%)
*Other AD, N (%)						
Bupropion	22 (2%)	24 (4%)	9 (5%)	30(1%)	47 (6%)	15 (3%)
Doxepin	2 (0.14%)	4 (0.6%)	0 (0%)	3(0.1%)	4 (0.5%)	3 (0.6%)
Imipramine	2 (0.14%)	0 (0%)	0 (0%)	2 (0.1%)	–	–
Mirtazapine	18 (1%)	24 (4%)	3 (2%)	30 (1%)	28 (3%)	18 (4%)
Selegiline	1 (0.1%)	0 (0%)	0 (0%)	–	–	–
Trazodone	34 (2%)	75 (11%)	32 (19%)	66 (3%)	77 (9%)	41 (8%)
Desipramine	–	–	–	–	1 (0.1%)	–

IQR, Interquartile Range (25% - 75%); IMT, immunosuppressive therapy; AD, antidepressant; CV, cardiovascular; aPLT w6m, antiplatelet therapy within 6 months; anti-coag w6m, anti-coagulation within 6 months

*Other non-SSRI or SNRI AD concomitantly administered.

Both cohorts had similar average 5-year Charlson scores among SSRI, SNRI and nonusers. Most patients with GCA were treated with glucocorticoids (steroids), whereas a very small percentage was treated with other immunosuppressive agents (tocilizumab or cyclophosphamide). 100% of patients with PMR were treated with oral glucocorticoids. Therefore, steroids were not used as an independent covariate in the most due to lack of contrast. The initial diagnosis of GCA or PMR, was made in the outpatient setting in more than 85% of the patients. The frequency of ischemic stroke, TIA, MI, and angina as well as prior history of CV events, and dual anti-platelet therapy within 6 months prior to the index date was slightly higher in the GCA cohort compared to the PMR cohort and varied within the groups of SSRI users, SNRI users and nonusers. A very small proportion of patients in both cohorts were on other antidepressants concurrently administered with SSRI or SNRI (**Table 1**).

### Frequency of cardiovascular events among SSRI, SNRI users and nonusers in patients with GCA and PMR

The occurrence of cardiovascular events in patients with GCA and PMR within the groups of SSRI users, SNRI users, as well as non-users, is presented in **Table 2**. In the GCA cohort, 174 (27%) SSRI users had incident cardiovascular disease as compared to 47 (28%) SNRI users and 277 (19%) nonusers; within the PMR cohort, incident cardiovascular disease was noted in 108 (13%) SSRI users in comparison to 71 (15%) SNRI users and 255 (11%) nonusers (**Table 2**).

**Table 2:** Frequency of CVE in SSRI/SNRI- and non-users in GCA and PMR.

GCA, n=2249	PMR, n=3906
*SSRI-users, n=653*	*SSRI-users, n=815*
Fluoxetine with CVE, n=33	Fluoxetine with CVE, n=13
Fluoxetine w/o CVE, n=69	Fluoxetine w/o CVE, n=107
Sertraline with CVE, n=73	Sertraline with CVE, n=60
Sertraline w/o CVE, n=185	Sertraline w/o CVE, n=369
Citalopram with CVE, n=51	Citalopram with CVE, n=17
Citalopram w/o CVE, n=159	Citalopram w/o CVE, n=109
Paroxetine with CVE, n=9	Paroxetine with CVE, n=11
Paroxetine w/o CVE, n=44	Paroxetine w/o CVE, n=47
Escitalopram with CVE, n=7	Escitalopram with CVE, n=6
Escitalopram w/o CVE, n=21	Escitalopram w/o CVE, n=73
Fluvoxamine with CVE, n=1	Fluvoxamine with CVE, n=1
Fluvoxamine w/o CVE, n=0	Fluvoxamine w/o CVE, n=2
Vortioxetine with CVE, n=0	*SNRI-users, n=487*
Vortioxetine w/o CVE, n=1	Venlafaxine with CVE, n=25
*SNRI-users, n=168*	Venlafaxine w/o CVE, n=102
Venlafaxine with CVE, n=27	Duloxetine with CVE, n=45
Venlafaxine w/o CVE, n=52	Duloxetine w/o CVE, n=312
Duloxetine with CVE, n=20	Milnacipran with CVE, n=0
Duloxetine w/o CVE, n=69	Milnacipran w/o CVE, n=1
*Non-SSRI or SNRI-users, n=1428*	Desvenlafaxine with CVE, n=1
Nonusers with CVE, n=277	Desvenlafaxine w/o CVE, n=1
Nonusers w/o CVE, n=1151	*Non-SSRI or SNRI-users, n=2604*
	Nonusers with CVE, n=255
	Nonusers w/o CVE, n=2349

### Association of SSRI and SNRI use with incidence of cardiovascular events in patients with GCA and PMR

The association between SSRI and SNRI use, and the incidence of CVE was then assessed in **Table 3**. In our adjusted main analysis that included nonusers and users of only one medication from the SSRI or SNRI group, we found venlafaxine and sertraline use was associated with a higher incidence of cardiovascular disease compared to nonusers, in both GCA and PMR subgroups. Patients with GCA who started using venlafaxine and sertraline within 6 weeks before diagnosis with use for at least a year were significantly more likely to have a higher incidence of cardiovascular events within 5 years from index date (adjusted OR for venlafaxine=2.44, 95%CI:1.23-4.84, p=0.01, and adjusted OR for sertraline=1.45, 95%CI:1.02-2.05, p=0.04). Similar results were observed in the PMR cohort (adjusted OR for venlafaxine =2.01, 95%CI:1.08-3.77, p=0.02, and adjusted OR for sertraline = 1.45, 95%CI:1.02-2.06, p=0.04). Citalopram was also associated with a higher cardiovascular incidence in the PMR cohort (adjusted OR=2.66, 95%CI:1.15-6.19, p= 0.02).

**Table 3:** Adjusted odds ratio of CVE for individual medication users in GCA and PMR within 5 years.

*CVE OR, (95% CI), p-value	
GCA	PMR
SSRI	SSRI
Fluoxetine 1.70 (0.98-2.94), 0.06	Fluoxetine 0.79 (0.38-1.67), 0.54
Sertraline 1.45 (1.02-2.05), 0.04	Sertraline 1.45 (1.02-2.06), 0.04
Citalopram 1.48 (0.98-2.23), 0.07	Citalopram 2.66 (1.15-6.19), 0.02
Paroxetine 0.77 (0.31-1.94), 0.58	Paroxetine 2.03 (0.81-5.11), 0.13
Escitalopram 2.36 (0.65-8.62), 0.20	Escitalopram 0.63 (0.23-1.70),0.35
Fluvoxamine -	Fluvoxamine -
Vortioxetine -	
SNRI	SNRI
Venlafaxine 2.44 (1.23-4.84), 0.01	Venlafaxine 2.01 (1.08-3.77), 0.02
Duloxetine 1.08 (0.56-2.11), 0.80	Duloxetine 1.25 (0.83-1.88), 0.28
Milnacipran -	Milnacipran -
Desvenlafaxine -	Desvenlafaxine -

*Adjusted for BMI, gender, age at the time of disease diagnosis, race, smoking, HTN, disease diagnosis in the outpatient or inpatient setting, and 5-year average Charlson’s score). For each regression, we included nonusers and users of only one medication.

To verify the robustness of our discovery we did a sensitivity analysis by including nonusers and users of all medications from both the SSRI and SNRI groups to perform a larger scale regression (**Supplementary Table 2**). In the adjusted MLR, venlafaxine use remained significantly associated with the higher odds of cardiovascular incidence (adjusted OR=2.18, 95%CI:1.31-3.63, p=0.002) followed by fluoxetine (adjusted OR=1.95, 95%CI:1.25-3.07, p=0.004), and sertraline (adjusted OR=1.51, 95%CI:1.10-2.06, p=0.01) in the GCA cohort. In the PMR cohort venlafaxine use continued to be associated with higher odds of cardiovascular disease (adjusted OR=2.34, 95%CI:1.46-3.74, p<0.001) followed by sertraline (adjusted OR=1.38, 95%CI:1.01-1.88, p=0.04).

We then applied Cox proportional hazard regression models to assess the HR of CVE among SSRI or SNRI users within 5 years in the GCA and PMR cohorts adjusting for comorbidity covariates. The HR for all cardiovascular events after 2:1 matching was significantly greater than 1 for both venlafaxine (p=0.03), and sertraline (p=0.02) in the GCA cohort, as well as in patients with PMR (p=0.003 and p=0.01 respectively) (**Table 4**). Paroxetine use was associated with the highest risk of developing cardiovascular disease in the GCA cohort (adjusted HR:2.41, 95%CI:1.09-5.33, p=0.03). However, due to the small number of patients under paroxetine, we believe the validity of this positive result needs further examination. Our results indicate that venlafaxine and sertraline users are at high risk of developing CVE in both GCA and PMR cohorts within 5 years of their disease course (**Table 4**).

**Table 4:** Adjusted hazard ratios of CVE in individual medication users in GCA and PMR after 2:1 matching within 5 years.

*CVE HR, (95%CI), p-value	
GCA	PMR
SSRI	SSRI
Fluoxetine 1.39 (0.80-2.43), 0.24	Fluoxetine 0.77 (0.35 -1.72), 0.53
Sertraline 1.43 (1.05-1.96), 0.02	Sertraline 1.51 (1.08-2.10), 0.01
Citalopram 1.24 (0.86-1.78), 0.24	Citalopram 0.87 (0.45-1.69), 0.69
Paroxetine 2.41 (1.09-5.33), 0.03	Paroxetine 0.81 (0.29-2.26), 0.68
Escitalopram 0.63 (0.16-2.48), 0.51	Escitalopram 1.99 (0.89-4.47), 0.09
Fluvoxamine -	Fluvoxamine -
Vortioxetine -	
SNRI	SNRI
Venlafaxine 1.77 (1.06-2.94), 0.03	Venlafaxine 2.39 (1.35-4.23), p=0.003
Duloxetine 0.86 (0.44-1.68), 0.67	Duloxetine 1.09 (0.74-1.62), 0.64
Milnacipran -	Milnacipran -
Desvenlafaxine -	Desvenlafaxine -

*Adjusted for BMI, gender, age at time of disease diagnosis, race, smoking, HTN, disease diagnosis in the outpatient or inpatient setting, and 5-year average Charlson’s score. For each HR, we included nonusers and users of only one medication.

Using all SSRI and SNRI medications in our Cox regression analysis, sertraline and venlafaxine use remained significantly associated with a high risk of CVE in the GCA cohort (adjusted HR:1.33, 95%CI:1.02-1.75, p=0.04, and adjusted HR:1.95, 95%CI:1.33-2.87, p<0.001 respectively), but not paroxetine use (adjusted HR:1.34, 95%CI:0.76-2.35, p=0.30) (**Supplementary Table 3**). For the PMR cohort, the use of sertraline and venlafaxine also continued to be associated with a higher risk of CVE (adjusted HR:1.43, 95%CI:1.08-1.91, p=0.01, and adjusted HR:2.09, 95%CI:1.39-3.15, p<0.001 respectively) (**Supplementary Table 3**).

We also compared the HRs among patients with SSRI or SNRI use to further eliminate any potential unmeasured confounders, to better validate the association between certain medications and observed higher incidence rates. These HRs are reported in **Supplementary Table 4**. For example, the HR of CVE among venlafaxine users in the PMR cohort is for comparing patients with venlafaxine use and PMR diagnosis to patients with any SSRI or SNRI use and PMR diagnosis. Non-users were not involved in this analysis. Interestingly, only venlafaxine use was associated with a higher risk of cardiovascular disease for both the GCA and PMR cohorts (**Supplementary Table 4**). The median duration of each SSRI or SNRI medication after the beginning of the study among the GCA and PMR cohorts is presented in **Supplementary Table 5**. The median duration of the SSRI medications varied between 299 to 454 days in the GCA cohort, whereas for the PMR patients was between 392 to 692. With regards to the SNRI medications, the median duration was between 364 to 478, and 214 to 720 days for the GCA and PMR cohorts respectively.

**Figure 1:**
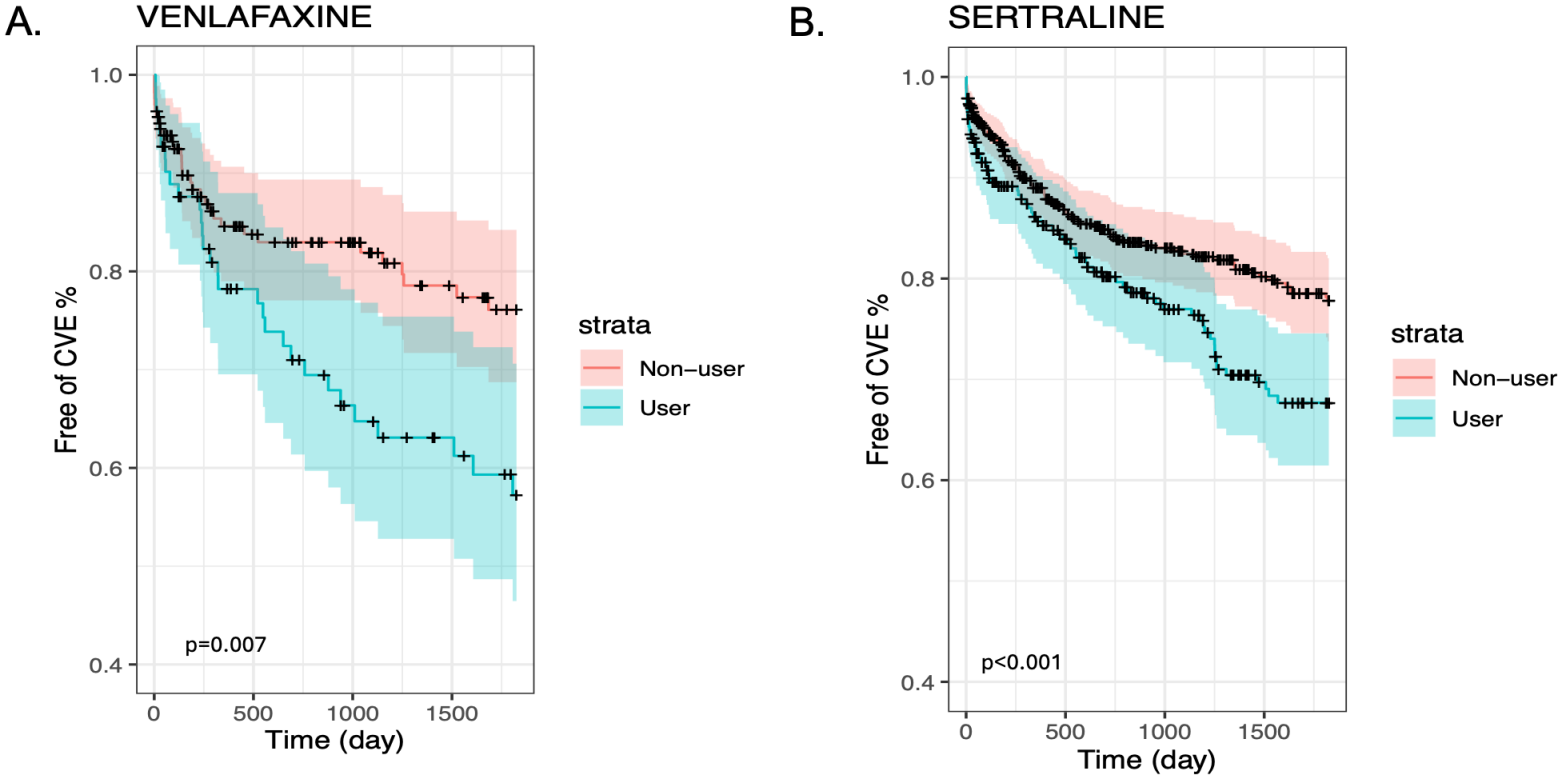
Kaplan-Meier plots for the time to CVE from the time of diagnosis of GCA stratified by medication user and non-user (blue and red lines respectively) after matching and including non-users and users of Venlafaxine **(A)** or Sertraline **(B)**.

**Figure 2:**
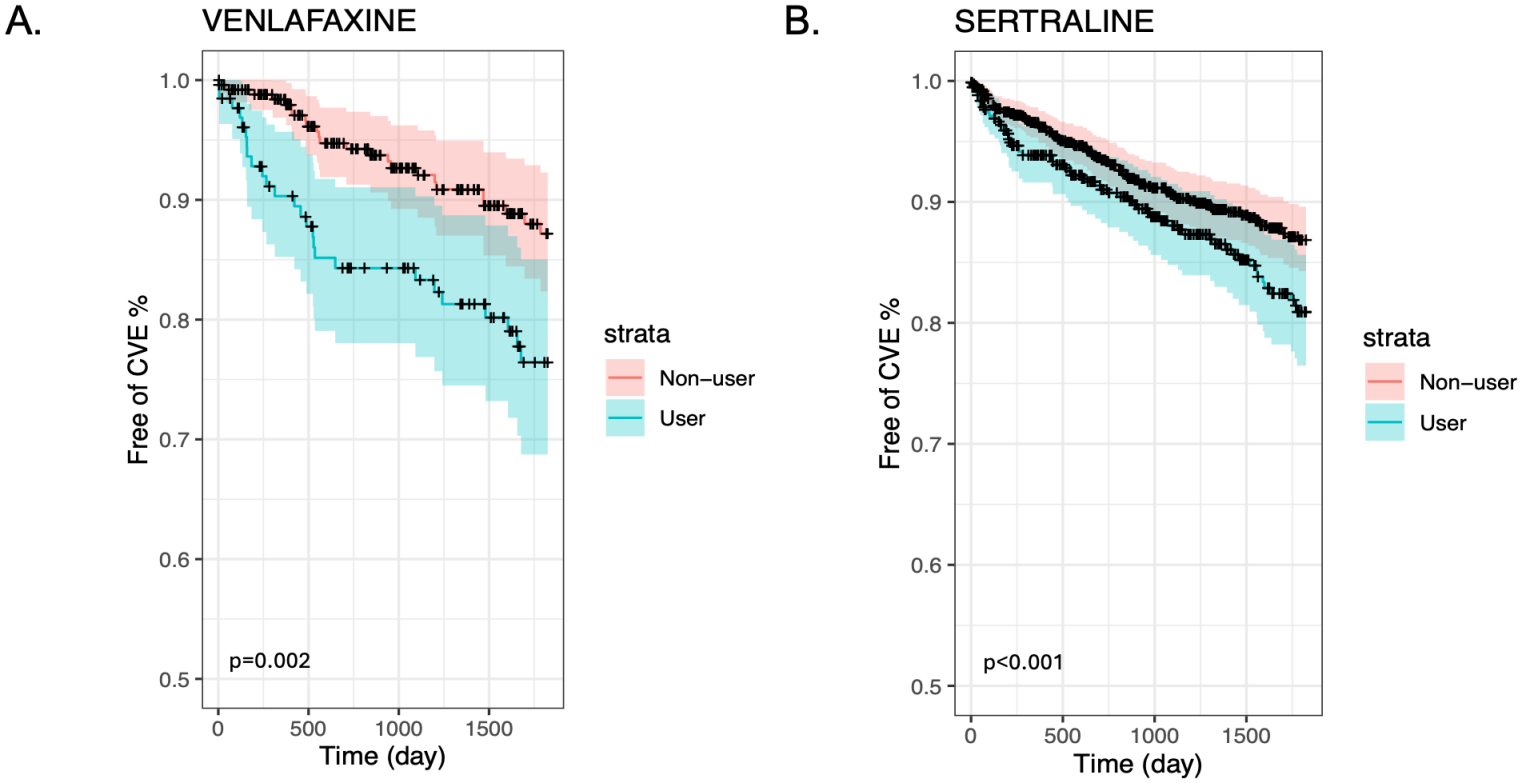
Kaplan-Meier plots for the time to CVE from the time of diagnosis of PMR stratified by medication user and non-user (blue and red lines respectively) after matching and including non-users and users of Venlafaxine **(A)** or Sertraline **(B)**.

We also present stratified Kaplan-Meier curves for the cardiovascular outcomes in GCA (**Figures 1A, B**) and PMR (**Figures 2A, B**). As shown in these figures, patients with GCA and PMR, stratified as venlafaxine or sertraline users, have a distinct time-to-event incidence of CVE events compared to non-users. The x-axis represents the time in days after each individual’s index date, whereas the y-axis shows the estimated probability of patients with GCA or PMR without developing cardiovascular events. For example, at 250 days after the diagnosis of GCA, the predicted probability of having CVE is around 12% for the nonusers, whereas the venlafaxine users have approximately 50% more chance of experiencing CVE in the GCA group (**Figure 1A**). As supplementary material we also present the predicted probability of experiencing cardiovascular outcomes among users of paroxetine and duloxetine compared to non-users in the GCA and PMR cohorts (**Supplementary Figures 1**, **2** respectively).

## Discussion

In this retrospective observational study, we observed a higher incidence of cardiovascular events among patients with GCA and PMR using the antidepressants venlafaxine or sertraline compared to nonuse. We also found a high risk of cardiovascular disease among venlafaxine and sertraline users in both the GCA and PMR cohorts. This observation may imply a harmful role of venlafaxine and sertraline use, increasing the risk for cardiovascular events among patients with GCA and PMR. We also recognize that depression which is common among patients with GCA and PMR (28, 29), is an independent risk factor for incident cardiovascular disease (30, 31).

To the best of our knowledge, there are no data on the impact of SSRI or SRNI use on cardiovascular burden in inflammatory diseases such as GCA and PMR. Patients with GCA are known to have increased mortality due to cardiovascular events including ischemic heart disease as shown in two independent studies conducted in northern (32) and southern Sweden (33) and verified by postmortem studies showing persistent vascular inflammation (34). However, in patients with PMR there was no association between cardiovascular events and mortality (35), despite some studies showing an increased risk of all types of cardiovascular events early in their disease course within the first six months after diagnosis (10).

An increasing amount of evidence suggests that platelet activation persists despite glucocorticoid therapy in patients with GCA and PMR (2, 3). Persistently elevated levels of von Willebrand factor that is produced by both endothelial cells and megakaryocytes (36, 37), as well as TSP-1 in the circulation of patients with GCA and PMR (3, 4), considered to be in clinical and biochemical remission, may indicate a constant procoagulant state. This prothrombotic effect could be modified by antidepressants that inhibit serotonin reuptake, in particular SSRI and SNRI, via depletion and/or decrease in levels of intraplatelet serotonin. Indeed, in the Sertraline Antidepressant Heart Attack Randomized Trial (SADHART) study, treatment with sertraline resulted in substantially less release of platelet (P-selectin, platelet factor 4, and thromboxane B2) and endothelial markers (vascular cell adhesion molecule-1 and E-selectin) as compared to placebo, in patients that suffered depression post-MI (38). However, evidence that these biochemical changes could have relevant cardiovascular significance remains unclear.

Interestingly, some studies indicate that sertraline use in depressed primates after an 18-month treatment period, may result in 4.9 times and 6.5 times higher coronary artery atherosclerosis extent compared to untreated depressed monkeys, and non-depressed monkeys respectively (39, 40), suggesting that both chronic sertraline use, and depression could be proatherogenic. Other preclinical studies have shown that chronic use of the SSRI fluoxetine enhanced the formation of atherosclerotic lesions in apolipoprotein E-deficient mice via increasing integrin activity on neutrophils and monocytes, potentially increasing the risk of cardiovascular events (41). In our sensitivity analyses, fluoxetine was also associated with a higher incidence of cardiovascular disease.

With regards to venlafaxine, it was shown that its use was associated with higher rates of stroke and TIA compared to other antidepressants among older people (42). Venlafaxine may promote inflammation and cytokine production, as its use is associated with elevated levels of tumor necrosis factor alpha (TNF- α), and interleukin-6 (IL-6), especially at higher doses of 150 mg daily due to its pro-norepinephrinergic effect (43, 44). IL-6 is an inflammatory cytokine with a known pro-atherogenic role (45). Of note, continued elevated plasma, and temporal artery tissue IL-6 levels reflecting ongoing vascular inflammation despite glucocorticoid therapy, has been reported in some patients with GCA (46). A subset of patients with PMR were also found to have elevated plasma IL-6 levels despite glucocorticoid therapy for a month (47). Further complicating matters, persistent systemic, and vascular inflammation in patients with PMR and GCA could be contributing to accelerated atherosclerosis and vascular remodeling leading to arterial stenosis and aneurysms (48, 49).

In a recent meta-analysis, SSRI use was associated with an increased risk of ischemic stroke (aOR 1.48; 95% CI 1.08–2.02), probably via its atherosclerotic effect on the cerebral vasculature (50). In our own study, we found that the aOR of cardiovascular events for sertraline use among patients with GCA and PMR was 1.46 and 1.45 respectively. In another study, SSRI use was also associated with an increased carotid intima-media thickness that is a predictor of myocardial infarction in a study of middle-aged veteran twins from the Vietnam Era Twin registry (51). Interestingly, in a propensity score-matched population-based study conducted in Canada there was a higher risk of acute MI, stroke, or cardiovascular related hospitalization among SNRI users compared to SSRI users within one year of drug initiation (52).

Our study has several limitations. Firstly, depression itself is a risk factor for cardiovascular events and could be a confounding factor by indication. Apart from indication bias, possible selection bias due to the retrospective nature of our study, and misclassification are also other limitations. We may have excluded patients with severe GCA or PMR disease as they may not have survived up to 150 days to be considered for immunosuppressive therapy or may have had a fatal cardiovascular event. Another limitation of our study is that the diagnosis of GCA and PMR was based on ICD codes. Also, inflammatory markers, histologic evidence of temporal arteritis for the diagnosis of GCA, imaging studies such as ultrasound of temporal arteries, magnetic resonance angiography and fluorodeoxyglucose (FDG)-positron emission tomography that is frequently used for diagnostic purposes, as well as disease severity and activity status, were not recorded for this study. Another limitation is that most patients with GCA and PMR were on glucocorticoid therapy, whereas only a very small proportion of patients with GCA were also on the IL-6 inhibitor tocilizumab, which could have a potential vascular protective role (45).

Additionally, information about the degree of physical activity and dietary preferences are not available for this study given its retrospective nature, and residual confounding is another limitation. Further, although our cohorts were large, the number of cardiovascular events was low which restricted the power of our study. Also, we did not use ICD codes for depression to identify patients who were depressed, and its severity, as we assumed that those on antidepressants had depression as an indication. Additionally, we did not use codes suggesting potential non medication adherence and/or codes associated with adverse effects of anti-depressants, resulting in discontinuation. Finally, it is unclear to what extent our results can be generalized to other autoimmune diseases such as rheumatoid arthritis and lupus that are independent risk factors for cardiovascular disease, or the general population in the presence of traditional risk factors, as there is a heavy representation of men in the military population.

However, our study has some strengths. This is the first large study that suggests that venlafaxine and sertraline use could be associated with a high incidence and risk of cardiovascular disease validated within two different disease cohorts, that share some common pathophysiological features. Another strength is that the reported adjusted OR and HR of cardiovascular events for venlafaxine and sertraline users demonstrated similar trends in both disease cohorts, enhancing further the robustness of our study findings. Although our findings suggest a cardio-harmful effect of venlafaxine and sertraline use, among these high-risk cardiovascular disease inflammatory conditions, our results need to be further validated by larger multicenter prospective clinical studies. Future research should explore the mechanisms by which specific antidepressants may influence cardiovascular health and assess the dose and duration of the effect of these pharmaceutical interventions on cardiovascular risk over time among patients with inflammatory disorders.

In conclusion, our study uncovers critical insights into how antidepressant pharmacotherapy, and particularly venlafaxine and sertraline can significantly modify cardiovascular risk profiles in patients with GCA and PMR. Given the widespread prescription of these medications for depression, it is imperative that clinicians and patients remain vigilant about the potential cardiovascular risks associated with their use. A careful assessment of the benefits versus risks is essential in guiding treatment decisions for these vulnerable populations in clinical practice. By prioritizing cardiovascular health, providers can improve clinical outcomes and quality of care for those affected by these inflammatory conditions.

## Data Availability

All data relevant to this study are included in this article. The dataset presented in this article is not readily available due to ethical/privacy restrictions. Requests to access the datasets should be directed to chris.gentry@va.gov.

## References

[1] Dejaco C Duftner C Buttgereit F Matteson EL Dasgupta B. The spectrum of giant cell arteritis and polymyalgia rheumatic: revisiting the concept of the disease. Rheumatol (Oxford). (2017) 56:506–15. doi: 10.1093/rheumatology/kew273 27481272

[2] Maugeri N Baldini M Rovere-Querini P Maseri A Sabbadini MG Manfredi AA. Leukocyte and platelet activation in patients with giant cell arteritis and polymyalgia rheumatica: a clue to thromboembolic risks? Autoimmunity. (2009) 42:386–8. doi: 10.1080/08916930902832629 19811309

[3] Michailidou D Kuley R Wang T Hermanson P Grayson PC Cuthberson D. Neutrophil extracellular trap formation in anti-neutrophil cytoplasmic autoantibody-associated vasculitis and large-vessel vasculitis. Clin Immunol. (2023) 249:109274. doi: 10.1016/j.clim.2023.109274 36878421 PMC10066833

[4] Michailidou D Johansson L Chapa J Wang T Chen J Lopez J. Mitochondrial-mediated platelet activation in patients with polymyalgia rheumatica. ACR Open Rheumatol. (2025) 7:e70021. doi: 10.1002/acr2.70021 40071558 PMC11897803

[5] Periayah MH Halim AS Mat Saad AZ. Mechanism action of platelets and crucial blood coagulation pathways in hemostasis. Int J Hematol Oncol Stem Cell Res. (2017) 11:319–27. PMC5767294 29340130

[6] Renga B Scavizzi F. Platelets and cardiovascular risk. Acta Cardiol. (2018) 72:2–8. doi: 10.1080/00015385.2017.1281560 28597734

[7] Amiri N De Vera M Choi HK Sayre EC Antonio A-ZJ. Increased risk of cardiovascular disease in giant cell arteritis: a general population-based study. Rheumatol (Oxford). (2016) 55:33–40. doi: 10.1093/rheumatology/kev262 26248811

[8] Greigert H Zeller M Putot A Steinmetz E Terriat B Maza M. Myocardial infarction during giant cell arteritis: A cohort study. Eur J Intern Med. (2021) 89:30–8. doi: 10.1016/j.ejim.2021.02.001 33610415

[9] Udayakumar PD Chandran AK Crowson CS Warrington KJ Matteson EL. Cardiovascular risk and acute coronary syndrome in giant cell arteritis: a population-based retrospective cohort study. Arthriits Care Res (Hoboken). (2015) 67:396–402. doi: 10.1002/acr.22416 PMC4310813 25074472

[10] Hancock AT Mallen CD Muller S Belcher J Roddy E Helliwell T. Risk of vascular events in patients with polymyalgia rheumatica. CMAJ. (2014) 186:E495–501. doi: 10.1503/cmaj.140266 PMC4162802 25070989

[11] Wozniak G Toska A Sandi M Mouzas O. Serotonin reuptake inhibitor antidepressants (SSRIs) against atherosclerosis. Med Sci Monit. (2011) 17:RA205–14. doi: 10.12659/MSM.881924 PMC3560505 21873959

[12] Pizzi C Mancini S Angeloni L Fontana F Manzoli L Costa GM. Effects of selective serotonin reuptake inhibitor therapy on endothelial function and inflammatory markers in patients with coronary heart disease. Clin Pharmacol Ther. (2009) 86:527–32. doi: 10.1038/clpt.2009.121 19641491

[13] Lozano PA Alarabi AB Garcia SE Boakye ET Kingbong HT Naddour E. The antidepressant duloxetine inhibits platelet function and protects against thrombosis. Int J Mol Sci. (2022) 23:2587. doi: 10.3390/ijms23052587 35269729 PMC8910021

[14] Blanchette CM Simoni-Wastila L Zuckerman IH Stuart B. A secondary analysis of a duration response association between selective serotonin reuptake inhibitor use and the risk of acute myocardial infarction in the aging population. Ann Epidemiol. (2008) 18:316–21. doi: 10.1016/j.annepidem.2007.11.004 18261924

[15] de Abajo FJ. Effects of selective serotonin reuptake inhibitors on platelet function: mechanisms, clinical outcomes, and implications for use in elderly patients. Drugs Aging. (2011) 28:345–67. doi: 10.2165/11589340-000000000-00000 21542658

[16] Sauer WH Berlin JA Kimmel SE. Selective serotonin reuptake inhibitors and myocardial infarction. Circulation. (2001) 104:1894–8. doi: 10.1161/hc4101.097519 11602490

[17] Glassman AH O'Connor CM Califf RM Swedberg K Schwartz P Bigger JT Jr. Sertraline treatment of major depression in patients with acute MI or unstab le angina. JAMA. (2002) 288:701–9. doi: 10.1001/jama.288.6.701 12169073

[18] Meier CR Schlienger RG Jick H. Use of selective serotonin reuptake inhibitors and risk of developing first time acute myocardial infarction. Br J Clin Pharmacol. (2001) 52:179–84. doi: 10.1046/j.0306-5251.2001.01426.x PMC2014522 11488775

[19] Michailidou D Zhang T Stamatis P Ng B. Risk of venous and arterial thromboembolism in patients with giant cell arteritis and/or polymyalgia rheumatica: A Veterans Health Administration population-based study in the United States. J Intern Med. (2022) 291:665–75. doi: 10.1111/joim.13446 34982490

[20] Michailidou D Zhang T Kuderer NM Lyman GH Diamantopoulos AP Stamatis P. Predictive models for thromboembolic events in giant cell arteritis: A US veterans health administration population-based study. Front Immunol. (2022) 13:997347. doi: 10.3389/fimmu.2022.997347 36439172 PMC9681825

[21] Lee H Tedeschi SK Chen SK Monach PA Kim E Liu J. Identification of acute giant cell arteritis in real-world data using administrative claims-based algorithms. ACR Open Rheumatol. (2021) 3:72–8. doi: 10.1002/acr2.11218 PMC7882520 33491920

[22] Fernandez-Avila DG Bernal-Macias S Rincon-Riano DN Gutierrez JM Rosselli D. Prevalence of polymyalgia rheumatica in Colombia: data from the national health registry 2012– 2016. Rheumatol Int. (2019) 39:1631–5. doi: 10.1007/s00296-019-04387-5 31327052

[23] Buitrago F. Cardiovascular events in patients with obesity: an observational study. Br J Gen Pract. (2010) 60:584–9. doi: 10.3399/bjgp10X515089 PMC2913738 20822691

[24] Ockene IS Miller NH. Cigarette smoking, cardiovascular disease, and stroke: a statement for healthcare professionals from the American Heart Association. Am Heart Assoc Task Force Risk Reduction. Circulation. (1997) 96:3243–7. doi: 10.1161/01.cir.96.9.3243 9386200

[25] de Boer RA Meijers WC van der Meer P van Veldhuisen DJ. Cancer and heart disease: associations and relations. Eur J Heart Fail. (2019) 21:1515–25. doi: 10.1002/ejhf.1539 PMC6988442 31321851

[26] Glasheen WP Cordier T Gumpina R Haugh G Davis J Renda A. Charlson comorbidity index: ICD-9 update and ICD-10 translation. Am Health Drug Benefits. (2019) 12:188–97. PMC6684052 31428236

[27] Fuchs FD Whelton PK. High blood pressure and cardiovascular disease. Hypertension. (2020) 75:285–92. doi: 10.1161/HYPERTENSIONAHA.119.14240 PMC10243231 31865786

[28] Martins-Martinho J Ponte A Dourado E Khmelinskii N Barreira SC Cruz-Machado AR. Anxiety and depression in patients with giant cell arteritis. Rheumatol Adv Pract. (2024) 8:rkae013. doi: 10.1093/rap/rkae013 38384323 PMC10879746

[29] Vivekanantham A Blagojevic-Bucknall M Clarkson K Belcher J Mallen CD Hider SL. How common is depression in patients with polymyalgia rheumatica? Clin Rheumatol. (2018) 37:1633–8. doi: 10.1007/s10067-017-3691-9 28573368

[30] Carney RM Freedland KE. Depression and coronary heart disease. Nat Rev Cardiol. (2017) 14:145–55. doi: 10.1038/nrcardio.2016.181 27853162

[31] Barlinn K Kepplinger J Puetz V IlIigens BM Bodechtel U Siepmann T. Exploring the risk-factor association between depression and incident stroke: a systematic review and meta-analysis. Neuropsychiatr Dis Treat. (2015) 11:1–14. doi: 10.2147/NDT.S63904 25565846 PMC4274141

[32] Uddhammar A Eriksson AL Nystrom L Stenling R Rantapää-Dahlqvist S. Increased mortality due to cardiovascular disease in patients with giant cell arteritis in northern Sweden. J Rheumatol. (2002) 29:737–42. 11950015

[33] Stamatis P Mohammad MA Gisslander K Merkel PA Englund M Turesson C. Myocardial infarction in a population-based cohort of patients with biopsy-confirmed giant cell arteritis in southern Sweden. RMD Open. (2024) 10:e003960. doi: 10.1136/rmdopen-2023-003960 38599652 PMC11015192

[34] Nordborg E Bengtsson B-A. Death rates and causes of death in 284 consecutive patients with giant cell arteritis confirmed by biopsy. BMJ. (1989) 299:549–50. doi: 10.1136/bmj.299.6698.549 PMC1837376 2507065

[35] Mykelbust G Wilsgaard T Jacobsen BK Gran JT. Causes of death in polymyalgia rheumatica. A prospective longitudinal study of 315 cases and matched population controls. Scand J Rheumatol. (2003) 32:38–41. doi: 10.1080/03009740310000382 12635944

[36] Cid MC Monteagudo J Oristrell J Vilaseca J Pallarés L Cervera R. Von Willebrand factor in the outcome of temporal arteritis. Ann Rheum Dis. (1996) 55:927–30. doi: 10.1136/ard.55.12.927 PMC1010347 9014589

[37] Uddhammar A Rantapää-Dahlqvist S Nilsson TK. Long-term follow up of von Willebrand factor and plasminogen activator inhibitor-1 in patients with polymyalgia rheumatica. Ann Rheum Dis. (1997) 56:698–9. doi: 10.1136/ard.56.11.698 PMC1752288 9462179

[38] Serebruany VL Glassman AH Malinin AI Nemeroff CB Musselman DL van Zyl LT. Platelet/endothelial biomarkers in depressed patients treated with the selective serotonin reuptake inhibitor sertraline after acute coronary events: the Sertraline AntiDepressant Heart Attack Randomized Trial (SADHART) Platelet Substudy. Circulation. (2003) 108(8):939–44. doi: 10.1161/01.CIR.0000085163.21752.0A 12912814

[39] Shively CA Register TC Appt SE Clarkson TB. Effects of long-term sertraline treatment and depression on coronary artery atherosclerosis in premenopausal female primates. Psychosom Med. (2015) 77:267–78. doi: 10.1097/PSY.0000000000000163 PMC4397139 25829239

[40] Shively CA Silverstein-Metzler M Justice J Willard SL. The impact of treatment with treatment with selective serotonin reuptake inhibitors on primate cardiovascular disease, behavior, and neuroanatomy. Neurosci Biobehav Rev. (2017) 74:433–43. doi: 10.1016/j.neubiorev.2016.08.037 PMC5366071 27590831

[41] Rami M Guillamat-Prats R Rinne P Salvermoser M Ring L Bianchini M. Chronic intake of the selective serotonin reuptake inhibitor fluoxetine enhances atherosclerosis. Arterioscler Thromb Vasc Biol. (2018) 38:1007–19. doi: 10.1161/ATVBAHA.117.310536 29567680

[42] Coupland CAC Dhiman P Barton G Arthur A Sach T Hippisley-Cox J. A study of the safety and harms of antidepressant drugs for older people: a cohort study using a large primary care database. Health Technol Assessment. (2011) 15:1366–5278. doi: 10.3310/hta15280 21810375

[43] Piletz JE Halaris A Iqbal O Hoppensteadt D Fareed J Zhu H. Pro-inflammatory biomakers in depression: treatment with venlafaxine. World J Biol Psychiatry. (2009) 10:313–23. doi: 10.3109/15622970802573246 19921973

[44] Kubera M Kenis G Bosmans E Kajta M Basta-Kaim A Scharpe S. Stimulatory effect of antidepressants on the production of IL-6. Int Immunopharmacol. (2004) 4:185–92. doi: 10.1016/j.intimp.2003.11.006 14996410

[45] Ridker PM Rane M. Interleukin-6 Signaling and anti-interleukin-6 therapeutics in cardiovascular disease. Circ Res. (2021) 128:1728–46. doi: 10.1161/CIRCRESAHA.121.319077.Epub 2021 May 17 33998272

[46] Weyand CM Fulbright JW Hunder GG Evans JM Goronzy JJ. Treatment of giant cell arteritis: interleukin-6 as a biologic marker of disease activity. Arthritis Rheumatol. (2000) 43:1041–8. doi: 10.1002/1529-0131(200005)43:5<1041:AID-ANR12>3.0.CO;2-7 10817557

[47] Weyand CM Fulbright JW Evans JM Hunder GG Goronzy JJ. Corticosteroid requirements in polymyalgia rheumatica. Arch Intern Med. (1999) 159:577–84. doi: 10.1001/archinte.159.6.577 10090114

[48] de Boysson H Aouba A. An updated review of cardiovascular events in giant cell arteritis. J Clin Med. (2022) 11:1005. doi: 10.3390/jcm11041005 35207277 PMC8878095

[49] Xu S Jiemy WF Boots AMH Arends S van Sleen Y Nienhuis PH. Altered plasma levels and tissue expression of fibroblast activation protein alpha in giant cell arteritis. Arthritis Care Res (Hoboken). (2024). doi: 10.1002/acr.25354 38685696

[50] Shin D Oh YH Eom CS Park SM. Use of selective serotonin reuptake inhibitors and risk of stroke: a systematic review and meta-analysis. J Neurol. (2014) 261:686–95. doi: 10.1007/s00415-014-7251-9 24477492

[51] Shah AJ Veledar E Shallenberger L Murrah N Jawed F Bremner D. Association of antidepressant medications with carotid intima media thickness in middle aged veteran twins. JACC. (2011) 57:E1588. doi: 10.1016/S0735-1097(11)61588-X

[52] Leong C Alessi-Severini S Enns MW Nie Y Sareen J Bolton J. Cerebrovascular, cardiovascular, and mortality events in new users of selective serotonin reuptake inhibitors and serotonin norepinephrine reuptake inhibitors. J Clin Psychopharmacol. (2017) 37:332–40. doi: 10.1097/JCP.0000000000000701 28383363

