# Supplementary material for: Association of SSRI and SNRI use with incidence of cardiovascular events in veterans with giant cell arteritis and polymyalgia rheumatica
Source: Front Immunol. 2025 Apr 24;16:1509941. doi: 10.3389/fimmu.2025.1509941 (PMC12058779; doi:10.3389/fimmu.2025.1509941)
Supplement: Supplementary file 1 [file DataSheet1.docx]

**Supplementary Table 1. ICD-9 and ICD-10 CM of diseases of interest**

| **Diagnosis** | **ICD-9 CM** | **ICD-10 CM** |
| --- | --- | --- |
| GCA | 446.5 | M31.6 |
| PMR | 725 | M35.3 |
| Ischemic stroke | 433-434, 436 | I67.81 |
| TIA | 435.9 | G45.9 |
| MI | 410 | I21, I22, I22.8, I22.9 |
| Angina | 362.31 | I200-201, I208-209, I240, I248-249 |
| Hypertension | 401.9 | I10 |
| Exclusive diagnosis (malignancy) | 199, 199.1, 415.11 | C80.11 |

GCA, giant cell arteritis; PMR, polymyalgia rheumatica; TIA, transient ischemic attack, MI, myocardial infarct; ICD-9CM, International classification of diseases Ninth Revision clinical modification code; ICD-10CM, International classification of diseases Tenth Revision clinical modification code.

**Supplementary Table 2. Adjusted odds ratio of CVE among SSRI and SNRI users in GCA and PMR within 5 years.**

| ***CVE OR, (95% CI),** **p-value** | |
| --- | --- |
| **GCA** | **PMR** |
| **SSRI** | **SSRI** |
| **Fluoxetine 1.95 (1.25-3.07), 0.004** | Fluoxetine 1.07 (0.57-1.98), 0.84 |
| **Sertraline 1.51 (1.10-2.06), 0.01** | **Sertraline**  **1.38 (1.01-1.88), 0.04** |
| Citalopram 1.26 (0.88-1.79), 0.20 | Citalopram 1.39 (0.82-2.36), 0.22 |
| Paroxetine 0.75 (0.36-1.58), 0.44 | Paroxetine 1.88 (0.93-3.80), 0.08 |
| Escitalopram 1.68 (0.70-4.06), 0.24 | Escitalopram 0.73 (0.31-1.72), 0.46 |
| Fluvoxamine - | Fluvoxamine - |
| Vortioxetine - |  |
| **SNRI** | **SNRI** |
| **Venlafaxine 2.18 (1.31-3.63), 0.002** | **Venlafaxine 2.34 (1.46-3.74), <0.001** |
| Duloxetine 1.14 (0.66-1.97), 0.64 | Duloxetine 1.30 (0.93-1.81), 0.13 |
| Milnacipran - | Milnacipran - |
| Desvenlafaxine - | Desvenlafaxine - |

*Adjusted for BMI, gender, age at the time of disease diagnosis, race, smoking, HTN, disease diagnosis in the outpatient or inpatient setting, and 5-year average Charlson’s score). As a supplementary sensitivity analysis of Table 3, we included nonusers and users of all groups of medications to perform a larger-scale regression.

**Supplementary Table 3. Adjusted hazard ratios of CVE among SSRI and SNRI users in GCA and PMR after 2:1 matching within 5 years.**

| ***HR (95%CI),** **p-value** | |
| --- | --- |
| **GCA** | **PMR** |
| **SSRI** | **SSRI** |
| Fluoxetine 1.27 (0.83-1.53), 0.28 | Fluoxetine 0.81 (0.41-1.58), 0.53 |
| **Sertraline 1.33 (1.02-1.75), 0.04** | **Sertraline**  **1.43 (1.08-1.91), 0.01** |
| Citalopram 1.13 (0.83-1.53), 0.44 | Citalopram 1.07 (0.61-1.87), 0.81 |
| Paroxetine 1.34 (0.76-2.35), 0.30 | Paroxetine 0.89 (0.40-2.01), 0.78 |
| Escitalopram 0.59 (0.19-1.85), 0.37 | Escitalopram 1.68 (0.94-3.00), 0.08 |
| Fluvoxamine - | Fluvoxamine  **-** |
| Vortioxetine - |  |
| **SNRI** | **SNRI** |
| **Venlafaxine** **1.95 (1.33-2.87), <0.001** | **Venlafaxine 2.09 (1.39-3.15), <0.001** |
| Duloxetine 0.23 (0.46-1.37), 0.41 | Duloxetine 1.09 (0.77-1.53), 0.61 |
| Milnacipran - | Milnacipran - |
| Desvenlafaxine - | Desvenlafaxine - |

*Adjusted for BMI, gender, age at time of disease diagnosis, race, smoking, HTN, disease diagnosis in the outpatient or inpatient setting, and 5-year average Charlson’s score. As a supplementary sensitivity analysis of Table 4, we included nonusers and users of all groups of medications.

**Supplementary Table 4. Adjusted hazard ratios of CVE of individual medication in comparison to other SSRI or SNRI medications in GCA and PMR within 5 years.**

| ***HR (95%CI),** **p-value** | |
| --- | --- |
| **GCA** | **PMR** |
| **SSRI** | **SSRI** |
| Fluoxetine 1.02 (0.65-1.59), 0.94 | Fluoxetine 0.60 (0.31-1.18), 0.14 |
| Sertraline 1.09 (0.81-1.46), 0.57 | Sertraline 1.16 (0.84-1.60), 0.35 |
| Citalopram 0.83 (0.60-1.15), 0.26 | Citalopram 0.79 (0.45-1.41), 0.43 |
| Paroxetine 1.12 (0.63-1.99), 0.68 | Paroxetine 0.68 (0.30-1.54), 0.35 |
| Escitalopram 0.47 (0.15-1.46), 0.19 | Escitalopram 1.33 (0.73-2.41), 0.35 |
| Fluvoxamine - | Fluvoxamine - |
| Vortioxetine - |  |
| **SNRI** | **SNRI** |
| **Venlafaxine 1.71 (1.15-2.53), 0.007** | **Venlafaxine 1.68 (1.10-2.58), 0.01** |
| Duloxetine 0.63 (0.36-1.10), 0.10 | Duloxetine 0.78 (0.54-1.13), 0.19 |
| Milnacipran - | Milnacipran - |
| Desvenlafaxine - | Desvenlafaxine - |

*Adjusted for BMI, gender, age at time of disease diagnosis, race, smoking, HTN, disease diagnosis in the outpatient or inpatient setting, and 5-year average Charlson’s score.

**Supplementary Table 5. Median duration of medication of interest in GCA and PMR after the beginning of study.**

| **Median duration of medication of interest (days)** | |
| --- | --- |
| **GCA** | **PMR** |
| **SSRI** | **SSRI** |
| Fluoxetine 454 | Fluoxetine 414 |
| Sertraline 384 | Sertraline 502 |
| Citalopram 393 | Citalopram 692 |
| Paroxetine 299 | Paroxetine 641 |
| Escitalopram 360 | Escitalopram 392 |
| *Fluvoxamine - | *Fluvoxamine - |
| Vortioxetine - |  |
| **SNRI** | **SNRI** |
| Venlafaxine 364 | Venlafaxine 720 |
| Duloxetine 478 | Duloxetine 214 |
| Milnacipran - | Milnacipran - |
| *Desvenlafaxine - | *Desvenlafaxine - |

* Not calculated because few patients were on Fluvoxamine and Desvenlafaxine
